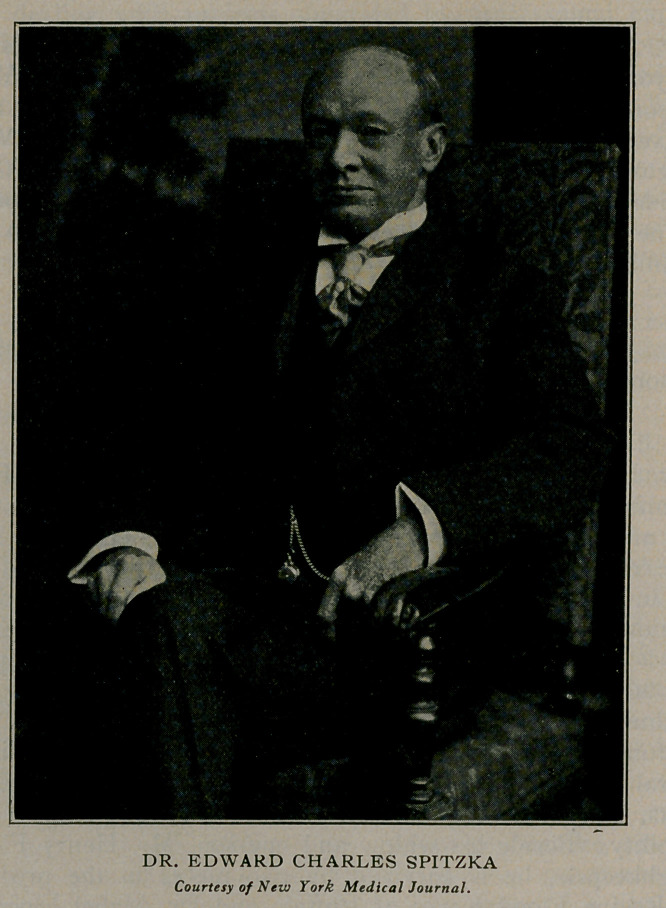# Dr. Edward Charles Spitzka

**Published:** 1914-02

**Authors:** 


					﻿Dr. Edward Charles Spitzka, University of New York 1873,
died at his home in Manhattan, January 13, 1914, of apoplexy,
aged 61. He had suffered for some time from necrosis of the
jaw. He was well known as a neurologist, and had been editor of
the American Journal of Necrology and President of the Amer
ican Neurologic Association and of the New York Neurologic
Society.
				

## Figures and Tables

**Figure f1:**